# The impact of the SARS-CoV-2 pandemic on cause-specific- mortality: a systematic literature review

**DOI:** 10.1093/eurpub/ckac131.192

**Published:** 2022-10-25

**Authors:** F Sanmarchi, F Esposito, E Adorno, MP Fantini, D Golinelli

**Affiliations:** DIBINEM, Department of Biomedical and Neuromotor Sciences, Bologna, Italy

## Abstract

**Background:**

Although investigating the patterns of COVID-19 excess mortality (EM) is relevant, understanding the effects of the pandemic on cause-specific mortality is even crucial and should also be assessed, as this metric allows for a more detailed analysis of the true impact of the pandemic. The aim of this systematic literature review is to estimate the impact of the pandemic on different causes of death, providing a quantitative and qualitative analysis of the phenomenon.

**Methods:**

We searched MEDLINE to identify studies that reported cause-specific mortality during the COVID-19 pandemic. We adopted several inclusion criteria: original article; assessed at least one cause-specific mortality during the pandemic; assessed causes of deaths using the ICD-10 classification; reporting of at least one of the following outcomes: cause-specific mortality estimates or cause-specific EM; full-length articles. Several relevant data were extracted (e.g. publication year, data stratification, territory, country income level, all-cause EM, and cause-specific mortality, etc.).

**Results:**

The search identified 548 articles. After title, abstract and full-text screening, we extracted relevant data from the final set of 14 articles. Cause-specific mortality was reported using different units of measurement. Only 9 studies reported the statistical significance and/or confidence intervals. The most frequently analyzed causes of death were cardiovascular diseases (n = 11), cancer (n = 7), diabetes (n = 6), and suicide (n = 5). We found very heterogeneous patterns of cause-specific mortality, for all the specific causes of deaths, except for suicide and road accident.

**Conclusions:**

The impact of the pandemic on cause-specific deaths has been very heterogeneous and the analyses conducted so far are not exhaustive. We advocate for the urgent need to find a consensus to define uniform methodological approaches to establish the true burden of the COVID-19 pandemic on non-COVID-19 mortality.

**Key messages:**

• We reviewed the body of literature to estimate the impact of the COVID-19 pandemic on different causes of death, and to provide a quantitative and qualitative analysis of the phenomenon.

• We did not identify unique patterns of cause-specific mortality due to too varied approaches in terms of disease classification and coding, and methodologies used for estimating mortality.

